# Shoulder dystocia—Still a feared complication. How can we improve?

**DOI:** 10.1111/aogs.14952

**Published:** 2024-08-22

**Authors:** Jens Fuglsang

**Affiliations:** ^1^ Department of Obstetrics and Gynecology Aarhus University Hospital Aarhus Denmark; ^2^ Department of Clinical Medicine Aarhus University Aarhus Denmark

Shoulder dystocia is a feared, yet well‐known, complication in Obstetrics. For the fetus and newborn, shoulder dystocia holds the potential of dramatic consequences. Perinatal death is the most severe complication but asphyxia, trauma to the central nervous system, to the brachial nerve plexus, and bone fractures may also leave permanent sequelae.^1^ For the birthing parent, perineal lesions and a traumatic experience of childbirth may have a lasting influence on life quality.

All obstetricians and midwives are aware of the risk of sudden shoulder dystocia in vaginal delivery and can, therefore, draw on an array of procedures to intervene in such cases. Trainees in obstetrics and gynecology and midwives will have learned various mnemotechnical acronyms to help in solving these emergency situations and, in many hospitals, team training programs exist with shoulder dystocia as one of the most frequent learning focuses. Individuals are trained in both hands‐on procedures as well as the coordination of team efforts in order to minimize damage incurred to the child and parent in case of shoulder dystocia. Hence, a great deal of awareness surrounds shoulder dystocia. However, the definition of shoulder dystocia is nevertheless difficult to pinpoint and there is some subjectivity involved in its definition.^1^

Four papers on shoulder dystocia are published in this issue of *Acta Obstetricia et Gynecologica Scandinavica*,^2^, ^3^, ^4^, ^5^ and these may boost our knowledge in managing and counseling on shoulder dystocia.

In the paper by Heinonen and colleagues, the manual maneuvers that midwives and obstetricians apply in case of a recognized shoulder dystocia are brought into play.^2^ In their study, Heinonen et al report that the use of obstetric maneuvers has increased during the study period from 2006 to 2015.^2^ At the same time, the rate of neonatal complications has declined. It, therefore, appears that the awareness and the treatment modalities for shoulder dystocia among birth attendants have increased, and this heightened awareness may have benefited in shoulder dystocia cases. Not unexpectedly, worse outcomes are demonstrated when the specific obstetric maneuvers directed toward shoulder dystocia are omitted and the higher the number of maneuvers the higher the risk of perineal lacerations,^2^ which is likely indicative of the most severe cases of shoulder dystocia.

This success nevertheless may come with a price. It is more difficult to ascertain whether the awareness of the risk of shoulder dystocia leads to a higher likelihood of ascribing a shoulder dystocia diagnosis to a delivery or whether obstetric maneuvers might be applied very early or even in cases that did not represent ‘real’ shoulder dystocia. It all comes down to the definition of shoulder dystocia.

Another take on the flow of actions in cases of shoulder dystocia is presented through the study by Hjorth‐Hansen et al.^3^ Here, real‐life video recordings of cases of shoulder dystocia were used for assessing the performance of the team of birth attendants in a situation of shoulder dystocia. The study was carried out in settings in which team training of personnel was an integrated part of being employed in the departments. Both technical and non‐technical skills in the real‐life situations were evaluated. For teams with a good technical performance, the non‐technical performance was often closely associated with the technical performance. In most cases, the awareness of manual procedures when shoulder dystocia was recognized was evaluated as good. However, one very important step, namely situational awareness with actual recognition of shoulder dystocia and the following step of alerting the patient in labor and attending staff had its challenges.^3^ Thus, defining, recognizing, and alarming around shoulder dystocia in real‐life cases is still not an easy task, despite regular training sessions for personnel.

The use of video recordings of real‐life situations are rare. Undoubtedly, there exists potential to improve team training sessions and the handling of real‐life shoulder dystocia cases through lessons learned by analysis of video recordings.

Prophylaxis, however, is better than treatment. In the papers by Rasmussen and colleagues^4^ and Jeppegaard and colleagues,^5^ recurrence rates for shoulder dystocia are reported in Norwegian and Danish settings.

A high risk of recurrence is demonstrated in the paper by Rasmussen and colleagues.^4^ Unsurprisingly, a large‐sized fetus is at an increased risk of shoulder dystocia, yet in more than 60% of shoulder dystocia cases, the birthweight of the newborn was less than 4500 g. Fetopelvic disproportion would be surmised and both maternal and fetal contributions to such disproportion would be expected. In the present study, it is even demonstrated that shoulder dystocia can be observed across generations with a higher risk of experiencing shoulder dystocia if the pregnant person has had a mother who has also experienced shoulder dystocia. Reasons for this finding remain speculative, yet maternal stature and the size of the pelvis and birth canal could be hypothesized as characteristics that to some degree are passed on to the next generation, not to mention overweight and genetic propensities to insulin resistance. A paternal contribution to shoulder dystocia was observed as well,^4^ most likely contributing to fetal size or proportions.

In the Danish study by Jeppegaard et al., the increased risk of recurrence of shoulder dystocia was found as well, especially if the mother had identifiable risk factors such as a fetus with a size of more than 4000 g or large‐sized fetus compared to the size of the newborn in the previous pregnancy that was complicated by shoulder dystocia.^5^ Additionally, signs of failure to progress as expected during vaginal delivery for a woman who had given birth before (eg oxytocin stimulation and operative delivery) were associated with recurrence of shoulder dystocia. These findings may not be very surprising, yet they remind caregivers that the choice of vaginal birth should be made only after thorough counseling of the pregnant individual. Similarly, birth attendants should be very aware of signs indicative of an increased risk of shoulder dystocia before and during vaginal delivery.

Women having a cesarean delivery in subsequent pregnancy were older, had a higher prevalence of diabetes, and had worse outcomes in the index pregnancy.^5^ The choice of a cesarean section in a subsequent pregnancy appear to be understandable.

In both the Norwegian and the Danish study, approximately 16% of women with a pregnancy following previous shoulder dystocia had a planned cesarean section.^4^, ^5^ Thus, overall, a previous episode of shoulder dystocia does not appear to lead to parents abstaining from having another pregnancy and the majority will attempt vaginal delivery in this next pregnancy. Still, though, we do not know enough about the dialog between the pregnant person and their caregivers neither in individuals aiming for a vaginal delivery nor in those scheduled for a planned cesarean section. What mattered for the pregnant person when considering delivery? What counseling was given? What was pivotal for the choice that was finally made? Both patients' and doctors' preferences may be of importance to the shared decision‐making.

Cases of shoulder dystocia will still be met at labor wards. As caregivers, we are obliged to provide the best possible help in these feared and often very dramatic situations. After an episode of shoulder dystocia, we should provide the patient (and partner) with the best possible advice regarding a subsequent pregnancy. The papers on shoulder dystocia in this issue of the AOGS are recommended as they provide relevant knowledge for both evaluating the risk of shoulder dystocia in a subsequent pregnancy and for continuous training programs in obstetrical skills at labor wards.
